# A novel and green nanoparticle formation approach to forming low-crystallinity curcumin nanoparticles to improve curcumin’s bioaccessibility

**DOI:** 10.1038/s41598-019-55619-4

**Published:** 2019-12-13

**Authors:** Ali Ubeyitogullari, Ozan N. Ciftci

**Affiliations:** Department of Food Science and Technology, University of Nebraska-Lincoln, Lincoln, NE 68588-6205 USA

**Keywords:** Chemistry, Engineering, Nanoscience and technology

## Abstract

Health-promoting effects of curcumin are well-known; however, curcumin has a very low bioavailability due to its crystalline structure. The main objective of this study was to develop a novel green nanoparticle formation method to generate low-crystallinity curcumin nanoparticles to enhance the bioavailability of curcumin. Nanoporous starch aerogels (NSAs) (surface area of 60 m^2^/g, pore size of 20 nm, density of 0.11 g/cm^3^, and porosity of 93%) were employed as a mold to produce curcumin nanoparticles with the help of supercritical carbon dioxide (SC-CO_2_). The average particle size of the curcumin nanoparticles was 66 nm. Impregnation into NSAs decreased the crystallinity of curcumin and did not create any chemical bonding between curcumin nanoparticles and the NSA matrix. The highest impregnation capacity was 224.2 mg curcumin/g NSA. Curcumin nanoparticles significantly enhanced the bioaccessibility of curcumin by 173-fold when compared to the original curcumin. The concentration of curcumin in the bioaccessible fraction was improved from 0.003 to 0.125 mg/mL by impregnation of curcumin into NSAs (42-fold). This is a novel approach to produce food grade curcumin nanoparticles with reduced crystallinity and maximize the utilization of curcumin due to increased bioaccessibility.

## Introduction

Curcumin (diferuloylmethane) is a polyphenolic compound which is mainly extracted from turmeric rhizome (*Curcuma longa*)^1^. It has been used in Ayurvedic medicine as a traditional pharmaceutical agent for thousands of years^2^. The health benefits of curcumin are well-documented; specifically, curcumin exhibits anticancer, antiviral, antioxidant, anti-inflammatory, antimicrobial, hypoglycemic, and antirheumatic properties^2,3^. Moreover, therapeutic applications of curcumin in the treatment of cancer, diabetes, cardiovascular diseases, neurodegenerative diseases, and gastrointestinal irritation are well established^2–4^. Human clinical trials have shown that curcumin is safe at high doses (12 g/day)^5^.

Although the health benefits of curcumin are well recognized, curcumin’s full potential has not yet been realized due to its low bioavailability. The bioavailability of curcumin is very low (~1%)^6^ due to its low water solubility, chemical instability and crystalline structure. Furthermore, incorporation of curcumin into foods is a major challenge from a technological and food quality standpoint because crude curcumin is a crystalline powder that is insoluble in water and poorly soluble in fats and oils, which affects the sensory and quality of the food product negatively.

Decreasing size of the bioactives is known to improve their solubilization and thereby their bioavailability; therefore, in recent years, there has been many research efforts to decrease the size of curcumin to increase its bioavailability^7,8^. Different approaches, such as formation of emulsions^9–15^, cyclodextrin complexes^16,17^, nanosuspensions by antisolvent precipitation^18^, and polysaccharide complexes^19–21^, and synthesis of colloidal particles^1^ have been used to improve the bioavailability of curcumin. However, the use of organic toxic solvents (i.e. chloroform) and surfactants limit their applications in food products; furthermore, most of those techniques produce liquid products which are difficult to handle and store. In addition, curcumin has low stability in aqueous medium^18^ which could be another disadvantage of some of those liquid formulations.

After exploring the new opportunities offered by supercritical fluid technology, and especially the green processing by SC-CO_2_ technology, there has been several efforts to decrease the size of curcumin using SC-CO_2_. So far, solid lipid particles by Particles Generated from Gas Saturated Solution (PGSS)^22^, micronization by Atomized Rapid Injection Solvent Extraction (ARISE)^23,24^, loading nanofibrous silk fibroin by Solution-Enhanced Dispersion via SC-CO_2_ (SEDS)^25^, and Precipitation by Pressure Reduction of Gas-Expanded Liquids (PPRGEL)^26^ have been employed to decrease the size of curcumin using SC-CO_2_. However, the difficulty in controlling the particle size, the use of organic solvents like acetone, and limited number of food-grade chemicals used during the production are still significant drawbacks of the current techniques. Therefore, there is a critical need for a new green approach to decrease the size and crystallinity of curcumin and in turn to improve curcumin’s bioavailability.

In our previous studies, low-crystallinity phytosterol nanoparticles were produced by impregnating a phytosterol-SC-CO_2_ solvato complex in the nanoporous starch aerogel (NSA) in a two-step process where first NSA was formed and then phytosterols were impregnated into the NSA using SC-CO_2_ ^27,28^. The size of the phytosterol particles formed in those studies ranged between 59 and 87 nm and the solubility of the phytosterols in water and gastrointestinal fluids was significantly improved. In this study, a novel single-step simultaneous NSA formation-nanoparticle formation method is introduced to form low-crystallinity curcumin nanoparticles.

Aerogels have been receiving a growing interest due to their outstanding properties (high surface area, nanoporous structure, and very low density) in biomedical and pharmaceutical industries. However, their food applications have not been explored yet. Starch is a great candidate to produce food-grade aerogels with nanoporous structure due to its gelatinization without any chemical cross-linker. Moreover, starch is a biodegradable, biocompatible, abundant, inexpensive and bio-based source for aerogel production^29^.

The main objective of this study was to develop a novel green nanoparticle formation approach to forming low-crystallinity curcumin nanoparticles that has improved bioaccessibility. The specific objectives were to: (a) develop a single-step simultaneous NSA formation-low-crystallinity curcumin nanoparticle formation process, (b) characterize curcumin nanoparticles impregnated in the NSAs, and (c) determine the bioaccessibility of the low-crystallinity curcumin nanoparticles using simulated digestion.

## Results and Discussion

This study introduces a novel green nanomanufacturing approach to generating low-crystallinity curcumin nanoparticles utilizing nanoporous starch aerogels in conjunction with SC-CO_2_ technology. The NSA formation process that was reported previously consisted of three major steps: gelatinization of wheat starch to form a hydrogel, replacing the water in the hydrogel with ethanol to produce an alcogel, and finally SC-CO_2_ drying of the alcogel to form an aerogel^29^. Gelatinization of starch results in swelling of the granules, amylose leaching and disruption of the ordered structure. On cooling, swollen granule sacs produce porous gel^30^. Solvent exchange was carried out prior to SC-CO_2_ drying because ethanol has higher solubility than water in SC-CO_2_ ^31^. Drying of the alcogel is critical to preserve the porous structure of the gel. Our previous studies have shown that air drying results in shrinkage of the structure and loss of all the pores due to high surface tension and capillary forces during drying^29^. However, SC-CO_2_ drying prevents the formation of liquid-vapor meniscus by eliminating the capillary forces in the pore walls (surface tension of the liquid in the pores)^32^. Therefore, SC-CO_2_ drying preserved the nanoporous structure of the alcogels and generated NSAs with outstanding properties: surface area of 60.4 ± 3.0 m^2^/g, pore size of 19.9 ± 2.5 nm, pore volume of 0.26 ± 0.01 cm^3^/g, density of 0.11 ± 0.00 g/cm^3^, and porosity of 92.8 ± 0.2%.

**Figure 1:**
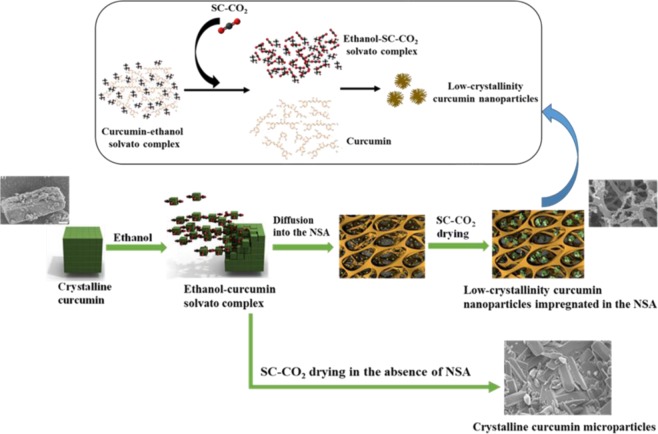
Illustration of the simultaneous NSA and low-crystallinity curcumin nanoparticle formation.

In this study, a simultaneous NSA formation-low-crystallinity curcumin nanoparticle formation process was developed (Fig. 1). NSAs were employed as a mold to produce curcumin nanoparticles in the nanopores of the aerogels. Ethanol-curcumin solvato complex diffused into the pores of NSAs during the last step of the solvent exchange. Then, the ethanol in the alcogel matrix was removed by SC-CO_2_ drying. During drying, curcumin was recrystallized from ethanol-curcumin solvato complex by anti-solvent action of the SC-CO_2_ in the pores of the NSAs which acted as a template and prevented the formation of long and well-ordered curcumin crystals (Fig. 1). As more ethanol was dissolved in the SC-CO_2_, curcumin started to precipitate from the ethanol-curcumin solvato complex in the pores of the NSA. The ethanol-curcumin solvato complex was confined in nanopores; meaning, there were limited amount of curcumin molecules in a small area; therefore, nanoparticles were formed. At the same time, a quick precipitation occurred in the nanopores, as a result, formation of well-ordered curcumin crystals was prevented. At 10 MPa and 40 °C, solubility of curcumin in SC-CO_2_ is very low (~4 * 10^−7^ g/L)^33^; therefore, while ethanol is dissolved in the SC-CO_2_ and carried outside, curcumin stays in the NSA. This novel nanomanufacturing method eliminated the additional SC-CO_2_ impregnation step after SC-CO_2_ drying which was the case in our previous phytosterol nanoparticle formation study^27^.

Few studies have reported formation of curcumin particles using SC-CO_2_ technology by PGSS, PPRGEL, ARISE, and SEDS processes^22–26^. PGSS technique uses CO_2_ as a solute. In PGSS process, SC-CO_2_ is dissolved in tristearin/soy phosphatidylcholine/DMSO/curcumin mixture and curcumin loaded solid lipid particles are produced by micronization of that mixture^22^. PPRGEL, a similar method to PGSS, is employed to produce curcumin particles by atomization of curcumin/acetone solution into water^26^. In addition, ARISE is based on atomization of curcumin mixture into a vessel pressurized with CO_2_. Feed solution is composed of curcumin/polyvinylpyrrolidone (PVP), hydroxypropyl-β-cyclodextrin (HPβCD), or both in organic solvents like methanol, ethanol or acetone^23^. Furthermore, SEDS process is based on precipitation of curcumin from acetone solution in SC-CO_2_. Curcumin-silk fibroin nanofibrous matrix is also produced using SEDS process^25^. However, the above-mentioned techniques do not provide a full control over the particle formation and result in particle agglomeration. None of those techniques reported any change in the crystallinity of the particles. In addition, the use of toxic solvents such as acetone and methanol makes those techniques not applicable in food industry. In this study, formation of low-crystallinity curcumin nanoparticles was reported for the first time.

### Morphology

**Figure 2:**
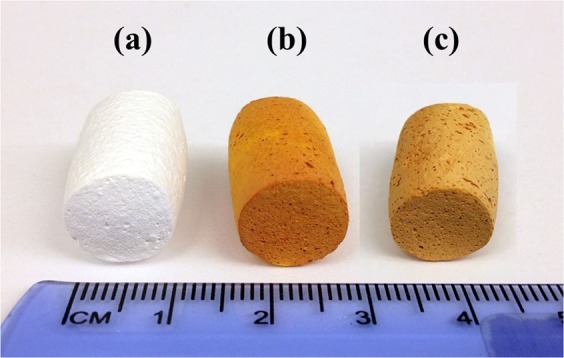
Pictures of (**a**) empty NSA and (**b**) CUR-NSA-60 °C and (**c**) CUR-NSA-RT.

Figure 2 depicts the pictures of the empty NSA (Fig. 2a), curcumin impregnated NSA at 60 °C (CUR-NSA-60 °C; Fig. 2b) and curcumin impregnated NSA at room temperature (CUR-NSA-RT; Fig. 2c). Both CUR-NSA-60 °C and CUR-NSA-RT had a uniform orange color in the matrix of aerogel monolith which indicates a successful diffusion of curcumin into the center of the monolith during solvent exchange. However, CUR-NSA-60 °C had darker orange color compared to CUR-NSA-RT due to higher impregnation capacity (explained in the impregnation capacity section).

**Figure 3:**
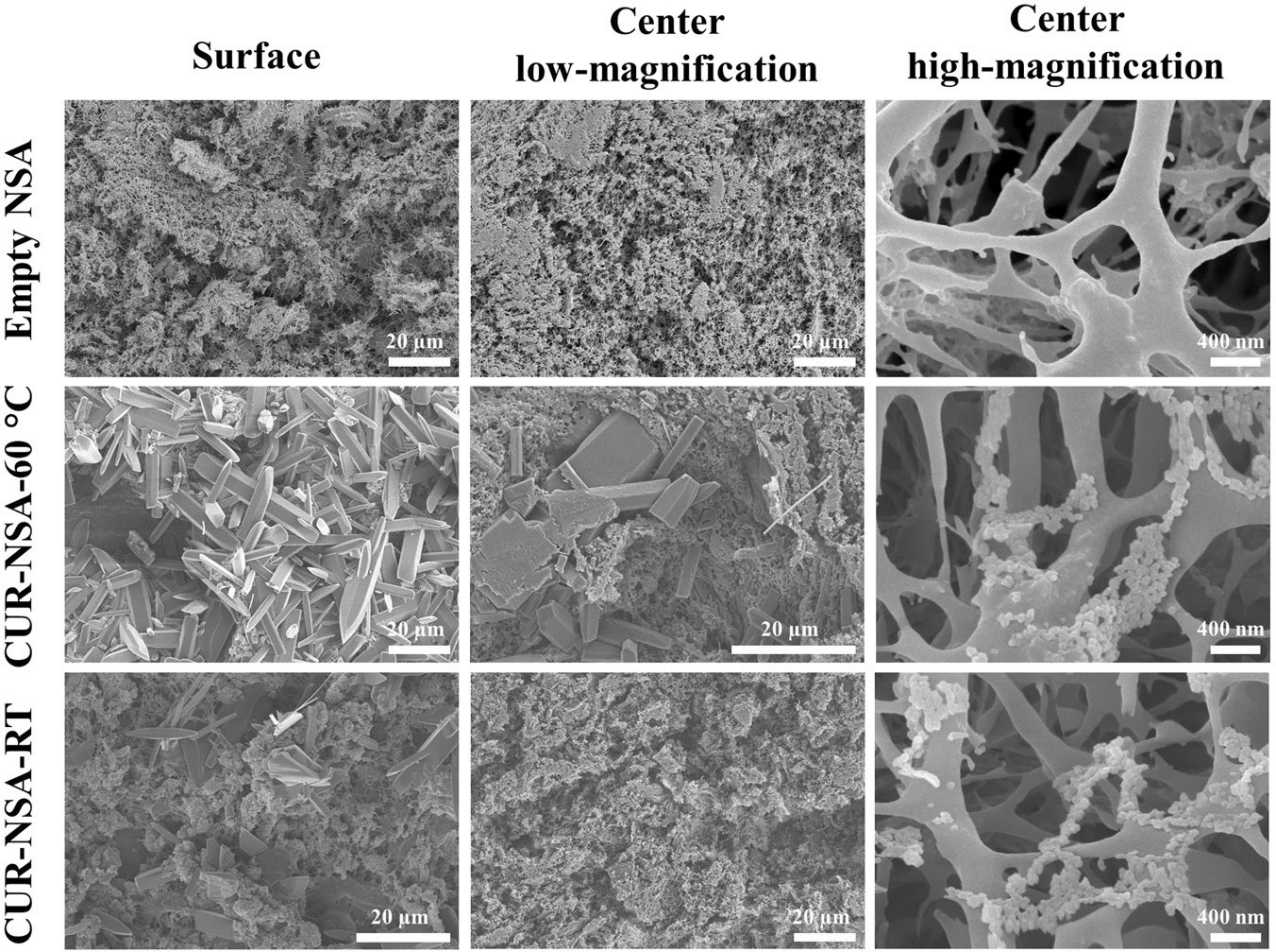
SEM micrographs of empty NSA, CUR-NSA-60 °C and CUR-NSA-RT.

SEM images of the empty NSA, CUR-NSA-60 °C and CUR-NSA-RT are presented in Fig. 3. Empty NSA had three-dimensional open porous structure. This network structure was not affected by curcumin impregnation. Curcumin plate-like crystals with lengths ranging between 5 and 50 µm formed a coat on the surface of the CUR-NSA-60 °C (Fig. 3). Although similar curcumin crystals were observed on the surface of the CUR-NSA-RT, they were fewer compared to that of the surface of the CUR-NSA-60 °C because curcumin was recrystallized on the surface of the NSAs from the bulk excess ethanolic curcumin solution and formed long curcumin crystals due to the absence of the NSA matrix. At the center of the CUR-NSA-60 °C monoliths, some curcumin crystals were still observed due to the high concentration of curcumin in the ethanolic curcumin solution. However, the formation of those long crystals in the center of the CUR-NSA-RT monoliths was prevented with lower concentration of ethanolic curcumin solution and the NSA matrix. Curcumin nanoparticles were observed in the center of both CUR-NSA-60 °C and CUR-NSA-RT monoliths at high magnification (Fig. 3). Curcumin nanoparticles impregnated in the NSA had a spherical morphology and their average particle size was 71 ± 8 and 66 ± 9 nm in the CUR-NSA-60 °C and CUR-NSA-RT, respectively. During SC-CO_2_ drying, curcumin nanoparticles showed a tendency to form agglomerates due to hydrophobic effect. Recently, Prasad *et al*. reported curcumin particle formation by PPRGEL process^26^. The mean particle size of curcumin varied between 0.4 µm and 30.8 µm and the particles had a plate-like structure. Besides, PGSS technique formed curcumin loaded solid lipid particle agglomerates with sizes over 100 µm^22^. Similarly, elongated thin curcumin crystals (~30 µm from ethanol and ~160 µm from acetone feed solutions) were fabricated with ARISE process^24^. On the other hand, SEDS process generated irregular-shaped agglomerated curcumin particles with the smallest average particle size of 325 nm^34^. However, those SC-CO_2_-based techniques did not provide a good control over the particle size and morphology. In this study, the formation of elongated curcumin crystals was prevented by the nanopores of the NSA.

### Crystallinity

**Figure 4:**
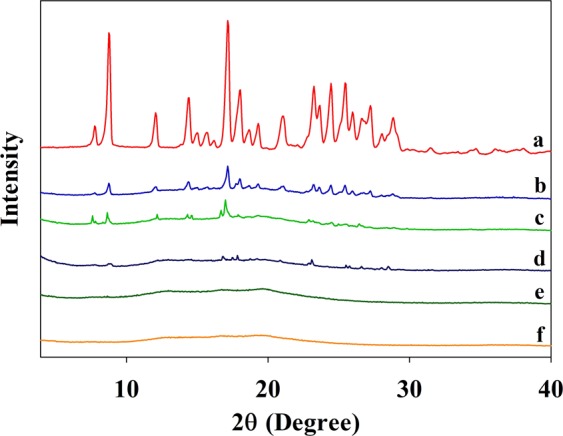
XRD patterns of (**a**) crude CUR, (**b**) physical mixture of crude CUR with empty NSA (224.2 mg crude CUR/g empty NSA), (**c**) CUR-NSA-60 °C, (**d**) physical mixture of crude CUR with empty NSA (14.4 mg crude CUR/g empty NSA), (**e**) CUR-NSA-RT, and (**f**) empty NSAs.

XRD patterns of the crude CUR (Fig. 4a), physical mixture of crude CUR with empty NSA (224.2 mg crude CUR/g empty NSA) (Fig. 4b), CUR-NSA-60 °C (Fig. 4c), physical mixture of crude CUR with empty NSA (14.4 mg crude CUR/g empty NSA) (Fig. 4d), CUR-NSA-RT (Fig. 4e), and empty NSA (Fig. 4f) are presented in Fig. 4. The mass ratios of crude CUR/empty NSA were determined according to the impregnation capacity of CUR-NSA-RT (see the impregnation capacity section). Crude curcumin had several strong characteristic peaks at 2*θ* = 8.8°, 12.1°, 14.3°, 17.2°, 18.0°, 21.1°, 23.1°, 24.4°, 25.5°, 27.2°, and 28.8°. Similar diffraction peaks were observed in the literature for crude curcumin powder^23,25^. These sharp peaks indicate the crystalline structure of crude curcumin. Empty NSAs had one broad peak, which means that empty NSAs were mainly in amorphous form due to disrupting the semi-crystalline structure of starch during gelatinization^30^. The XRD pattern of the physical mixture of the crude curcumin with the empty NSA had the same characteristic diffraction peaks with the crude curcumin (Fig. 4a,b,d). However, the intensity of these peaks was much lower when curcumin was impregnated into NSA at the same mass ratio (Fig. 4c,e), which shows the reduced crystallinity of the curcumin particles impregnated in the NSA. Curcumin in the CUR-NSA-60 °C had relatively higher crystallinity than that in CUR-NSA-RT due to higher amount of curcumin crystals on the surface (Figs. 3 and 4c,e). Reduced crystallinity enhances the dissolution rate of the water-insoluble bioactives due to the increase in the lattice free energy^35,36^; therefore, it is expected that the bioavailability of the low-crystallinity bioactives will be higher compared to higher-crystallinity bioactives.

### Chemical interaction between the curcumin and the NSA

**Figure 5:**
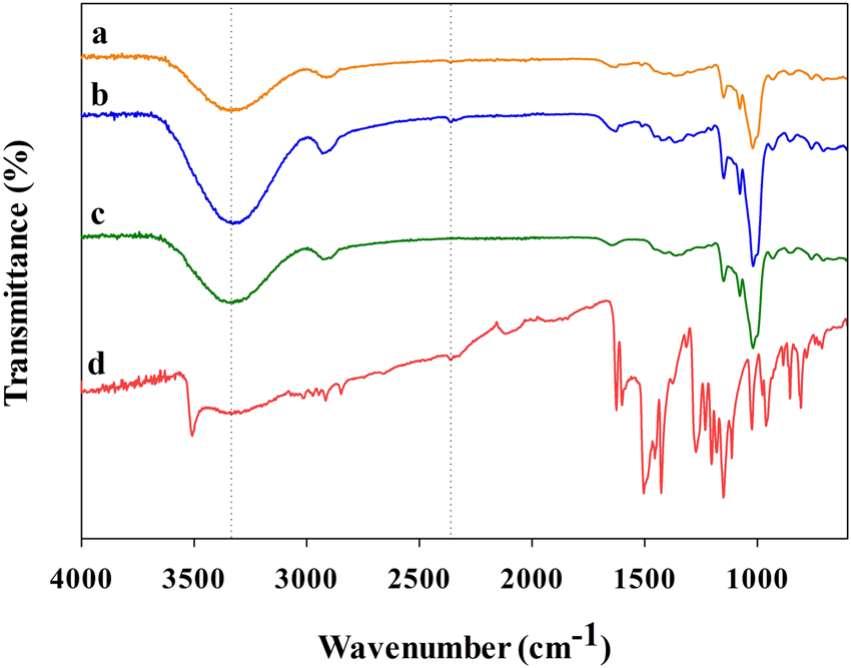
ATR-FTIR spectra of the (**a**) CUR-NSA-RT, (**b**) CUR-NSA-60 °C, (**c**) empty NSA, and (**d**) crude CUR.

Figure 5 shows the absence of possible chemical interactions between the low-crystallinity curcumin nanoparticles and the NSA. The FTIR results revealed that there was no shifting on the location of the characteristic peaks of crude curcumin, meaning there was no interaction between the curcumin particles and the NSA (Fig. 5a,b,d). Empty NSA (Fig. 5c) showed characteristic band between 3660 and 2990 cm^−1^ for the O–H stretching; and exhibited peaks at 2900 cm^−1^ for C–H stretching vibrations, 1150 cm^−1^ for C–O–C glucosidic bridge, and 1180 and 1020 cm^−1^ corresponding to C–C and C–O stretching vibrations which are consistent with the previous reports^27,37^. The FTIR spectrum of crude curcumin (Fig. 5d) exhibited a broad peak between 3450 and 3090 cm^−1^ and a sharp peak at 3510 cm^−1^ for O–H stretching; and peaks at 1625 cm^−1^ for mixed C=O and C=C vibrations, 1602 cm^−1^ for aromatic ring stretching vibrations, 1504 cm^−1^ for C–O and C–C vibrations, 1427 cm^−1^ for olefinic C-H bending vibrations, 1272 cm^−1^ for aromatic C–O stretching vibrations, and 1025 cm^−1^ for C–O–C stretching vibrations^19,38^. Even though impregnated curcumin nanoparticles tended to recrystallize close to each other (Fig. 3), having no interaction between the impregnated curcumin and NSA indicates easy release of curcumin into water or gastrointestinal tract after oral administration.

### Impregnation capacity

**Figure 6:**
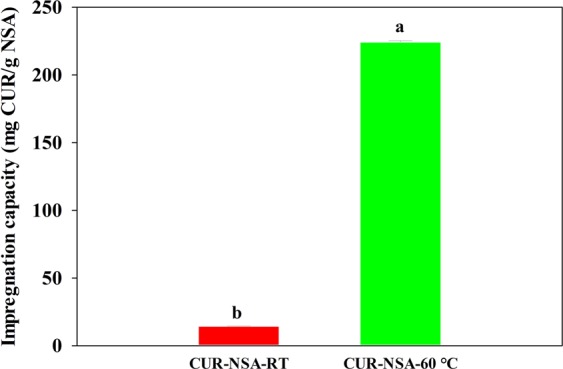
Curcumin impregnation capacities of the NSA generated at different impregnation temperatures.

Impregnation capacity of the CUR-NSA-RT, where impregnation was performed at room temperature (21 °C), was 14.4 mg CUR/g NSA (Fig. 6). When temperature of the impregnation increased to 60 °C (CUR-NSA-60 °C), the impregnation capacity significantly increased to 224.2 mg CUR/g NSA (Fig. 6) (p < 0.05). Impregnation capacity was basically based on the solubility of curcumin in ethanol. As the temperature of ethanol was raised from 21 to 60 °C, the solubility of curcumin in ethanol increased from 4.4 ± 0.2 to 17.2 ± 0.3 mg curcumin/mL ethanol. The increase in the solubility was not exactly at the same order with the increase in the impregnation capacity; a 3.9-fold increase in the solubility of curcumin in ethanol resulted in 15.6-fold increase in the impregnation capacity. The reason for a drastic increase in the impregnation capacity at 60 °C was the crystallization of curcumin in the saturated ethanolic curcumin solution on the surface of the NSA (Fig. 3) due to temperature drop to 40 °C during SC-CO_2_ drying.

Zou *et al*. investigated the use of excipient emulsions to improve the solubility and bioaccessibility of curcumin^11^. Emulsions were formed by mixing an aqueous phase of 1% (w/w) Tween 80 buffer solution with corn oil. The emulsions were passed through a microfluidizer to prepare small particle size emulsions. Then, curcumin was added into the emulsions at a ratio of 0.3 mg curcumin/mL emulsion which is much lower than the impregnation capacity obtained in this study. The maximum amount of curcumin incorporated into the emulsions was limited by the solubility of curcumin in corn oil (~3.2 mg/mL)^39^. One more drawback of the emulsion formulations is that the oil phase (where curcumin is dissolved) in the emulsions is in contact with an aqueous phase which increases the chemical degradation of curcumin because of low stability of curcumin in an aqueous medium^18^.

In another study, zein-curcumin colloidal particles were synthesized using an antisolvent precipitation method^1^. Solvents like methanol, ethanol and isopropyl alcohol were used with and without stabilizers (sodium caseinate or PVP K-30) to dissolve zein-curcumin mixture. Loading capacity of zein-curcumin colloidal particles was lower than that of CUR-NSA-60 °C and ranged between 1.6 and 4.1% (w/w) depending on the ratio of zein and curcumin used. In that study, 0.05% (w/w) Tween 80 was required to have a dispersion of the colloidal particles which limits the application of those particles in foods. Furthermore, soy soluble polysaccharides were used as nanocarrier for curcumin where curcumin was first dissolved in ethanol and then added to an aqueous mixture of a polysaccharide solution^20^. That study reported the highest loading capacity was 4.49 mg CUR/g polysaccharide, which is fifty times lower than the highest impregnation capacity (224.2 mg CUR/g NSA) obtained in this study.

### Simulated digestion

**Figure 7:**
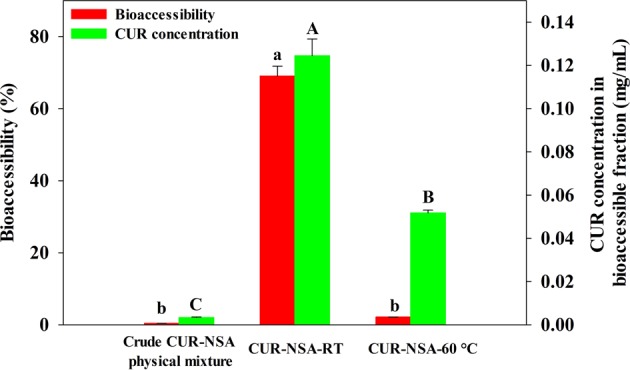
Bioaccessibility and concentration of curcumin in the samples after simulated digestion. Different lower-case letters show statistical significance in the bioaccessibility of curcumin (P < 0.05) and different capital letters show statistical significance in curcumin concentration in the bioaccessible fraction of the samples (P < 0.05).

Low-crystallinity curcumin nanoparticles, both CUR-NSA-RT and CUR-NSA-60 °C, had significantly higher *in vitro* bioaccessibility compared to crude CUR (Fig. 7). The bioaccessibility of crude curcumin was only 0.4%, which was expected due to its low water solubility and crystalline structure. The highest bioaccessibility of curcumin was obtained with impregnation at room temperature (CUR-NSA-RT) (p < 0.05). The bioaccessibility of the CUR-NSA-RT (69.1%) was 173-fold higher than that of crude curcumin (Fig. 7). Although the bioaccessibility of crude CUR-NSA physical mixture (0.4%) and CUR-NSA-60 °C (2.2%) were not significantly different (p > 0.05), CUR-NSA-60 °C resulted in a significantly higher concentration of curcumin in the bioaccessible fraction (0.052 mg/mL) than the crude curcumin (0.003 mg/mL). The highest curcumin concentration in the bioaccessible fraction was achieved with CUR-NSA-RT as 0.125 mg/mL, which was 42 times higher than that of crude curcumin. Although the impregnation capacity of CUR-NSA-60 °C was significantly higher than that of CUR-NSA-RT (Fig. 6), both the bioaccessibility and curcumin concentration in the bioaccessible fraction of CUR-NSA-60 °C were significantly lower (p < 0.05). As discussed before, dissolving curcumin at 60 °C formed a higher concentration ethanolic curcumin solution compared to the one prepared at room temperature. When the hydrogel was immersed in the curcumin solution, there were more curcumin on the surface of the alcogel. When the alcogel was dried, the curcumin on the surface of the alcogel crystallized on the surface of the aerogel. Because of the high concentration of the solution prepared at 60 °C, there were significantly higher amount of well-ordered plate-like curcumin crystals on the aerogel (Fig. 3); therefore, a higher impregnation capacity was obtained. However, those well-ordered plate-like crystals were not bioaccessible after simulated digestion due to their large size and well-ordered structure, which led to a lower bioaccessibility and lower concentration in the bioaccessible fraction of CUR-NSA-60 °C.

**Figure 8:**
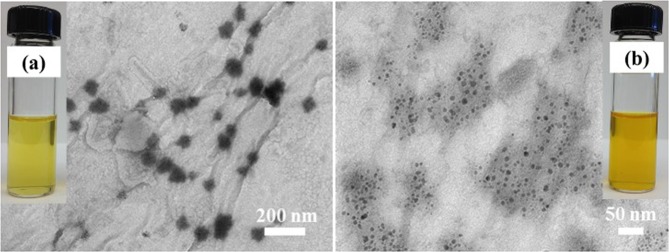
TEM images and gross appearance (inset images) of the bioaccessible fractions of (**a**) physical mixture of crude CUR with empty NSA, and (**b**) CUR-NSA-RT.

Figure 8 depicts the pictures and TEM images of the bioaccessible fractions obtained after simulated digestion of the physical mixture of crude CUR with empty NSA and CUR-NSA-RT. The color of the bioaccessible fraction of CUR-NSA-RT (Fig. 8b) was darker compared to crude curcumin (Fig. 8a), indicating a higher curcumin concentration. TEM images of the bioaccessible fractions revealed the particle size of the curcumin particles in the bioaccessible fractions. The size of the curcumin particles in the bioaccessible fraction of CUR-NSA-RT (10 ± 1 nm) was significantly smaller than that of crude curcumin (67 ± 9 nm) (p < 0.05). The smaller curcumin particles are expected to have higher absorption after digestion because of the higher permeability trough biological barriers^40,41^. To illustrate, silver nanoparticles (10, 20, 75 and 110 nm) were investigated at the same concentration for their epithelial permeability using T84 human colonic epithelial cells, and the particle size of 10 nm resulted in the highest permeability^42^. Therefore, it is expected that smaller curcumin particles in the bioaccessible fraction will lead to a higher bioavailability of curcumin due to higher absorption rates.

It is necessary to determine the stability and solubility of the curcumin particles in digestion fluids with changing environment like pH, and ionic strength in addition to physical characterization after particle formation. Simulated digestion is a useful tool to determine performance of the curcumin particles under gastrointestinal conditions in a short time^43^. Despite of the efforts to form curcumin particles using SC-CO_2_ technology (PGSS, ARISE, SEDS and PPRGEL), none of those studies determined the bioaccessibility of their products. Among the other methods, Aditya *et al*. investigated the oral bioavailability of β-lactoglobulin-curcumin nanosuspensions using Caco-2 cells^18^. The bioavailability of crude curcumin was ~2.7% after 3 h incubation and it was increased to ~3.1% with crystalline curcumin nanosuspensions without β-lactoglobulin. The highest bioavailability was achieved with β-lactoglobulin-curcumin nanosuspension as ~4%. However, those formulations are pH dependent and may not be stable in certain type of foods and protein-curcumin nanosuspensions may agglomerate due to different pHs throughout gastrointestinal tract. The bioavailability of curcumin nanoparticles obtained in this study is expected to be higher than that of β-lactoglobulin-curcumin nanosuspensions because curcumin nanoparticles were smaller (66 nm versus 150–175 nm) and had less crystallinity. Nanoemulsions, zein nanosuspensions and nanoliposomes were also explored to improve the bioaccessibility of curcumin^12^. Those formulations were prepared by injecting ethanol/oil solution into Tween 80 and phosphate buffer saline (PBS) buffer solution mixture. Loading capacities were 11.7, 3.1 and 0.4% (w/w) for protein, phospholipid and lipid nanoparticle suspensions, respectively. Although loading capacities of protein and phospholipid nanoparticle suspensions were relatively high, but still lower than the impregnation capacity obtained in this study (18.3%, w/w), curcumin was not stable in those formulation; 59–79.2% of the curcumin degraded during simulated digestion. A high bioaccessibility (91.8%) was reported for lipid nanoparticle suspensions (nanoemulsions) but the concentration in the bioaccessible fraction was only 109 µg/mL^12^. The high bioaccessibility (91.8%) obtained with lipid nanoparticle suspension was a reason of low loading capacity (0.4%, w/w). In another study by the same research group, the bioaccessibility of curcumin was around 70% when excipient emulsions were used and the highest concentration of curcumin in the bioaccessible fraction was ~90 µg/mL^11^. Those emulsion studies do not have a comparison with the commercial curcumin, therefore the rate of improvement in the bioaccessibility of crude curcumin is not clear.

Li *et al*. developed curcumin loaded nanoemulsions that were covered by chitosan in order to protect curcumin from degradation^10^. The prepared nanoemulsions had a curcumin concentration of 0.548 mg/mL. Although chitosan coating was effective in reducing the degradation of curcumin during thermal and UV irradiation treatments, the bioaccessibility of curcumin was slightly (less than 5% compared to nanoemulsions) decreased with middle and high molecular weight chitosan coating of the nanoemulsions.

In another approach, free lipid droplets, lipid-loaded alginate beads and lipid-loaded carrageenan beads were loaded with curcumin^21^. The size of the alginate beads ranged between 2 and 3 mm whereas carrageenan beads had a diameter in the range of 3–4 mm. Curcumin concentration in the bioaccessible fraction after simulated digestion was ~35, ~15 and ~8 µg/mL for free lipid droplets, loaded alginate beads and loaded carrageenan beads, respectively. The current study achieved at least 3.6-fold higher curcumin concentration in the bioaccessible fraction. In another study, bioaccessibility of soy soluble polysaccharides and curcumin complexes was determined by a sequential *in vitro* gastric and intestinal digestion^20^; however, oral phase digestion was missing in that study^44^. They reported the bioaccessibility of crude curcumin as 24.8 and 34.8% at pH 7.0 and 4.0, respectively. The bioaccessibility of curcumin was improved up to 76.8 and 82.8% by soy soluble polysaccharide-curcumin complexes at pH 7.0 and 4.0, respectively, meaning a 3-fold increase in the bioaccessibility of curcumin.

## Conclusions

This study has described a novel nanomanufacturing method utilizing SC-CO_2_ technology and NSAs to fabricate low-crystallinity curcumin nanoparticles to improve the bioavailability of curcumin. Curcumin nanoparticles were in spherical shape and their average size was 66 nm. The crystallinity of curcumin was decreased by impregnation into NSAs which enhances the dissolution rate of curcumin in the digestive fluids and consequently improves its bioavailability. There was no chemical bonding between impregnated curcumin nanoparticles and the NSA, therefore release mechanisms of curcumin nanoparticles is expected to improve. The highest impregnation capacity was obtained at an impregnation temperature of 60 °C as 224.2 mg curcumin/g NSA. However, the bioaccessibility of curcumin was maximized with an impregnation at room temperature. The highest bioaccessibility and concentration of curcumin in the bioaccessible fraction were 69.1% and 0.125 mg/mL, respectively. Curcumin nanoparticles had 173-fold higher bioaccessibility than crude curcumin and the concentration of curcumin in the bioaccessible fraction was significantly improved by impregnation into NSAs (42-fold).

This is a new green nanomanufacturing technology to form low-crystallinity bioactives. This novel approach has the potential to (i) enhance the efficacy of various bioactive compounds; (ii) allow food manufacturers to incorporate lipophilic bioactive compounds into foods to produce health and wellness improving foods in a green and simple way; (iii) increase the cost-benefit ratio of various bioactive compounds; (v) make the handling, storage & transportation of bioactives easy, because NSA formulation is a dry powder whereas most of the current lipophilic bioactive delivery systems are liquid.

## Methods

### Materials

Crude curcumin (>98% purity) was acquired from Acros Organics (NJ, USA). The composition of crude curcumin was determined in the lab by high-performance liquid chromatography (HPLC) and found to be 2.0 ± 0.3% bisdemethoxycurcumin, 15.0 ± 0.4% demethoxycurcumin, and 83.0 ± 0.7% curcumin. Liquid CO_2_ (99.99% purity) was obtained from Matheson Tri-Gas, Inc. (NE, USA).

The α-Amylase enzyme (from *Bacillus subtilis*, 160,000 BAU/g) was purchased from MP Biomedicals (OH, USA). Pepsin (3,616 U/mg protein), pancreatin (neutral protease: 208 USP U/mg solid; α-Amylase: 223 U/mg solid; lipase: 38.5 USP U/mg solid), pancreatic lipase (419 U/mg protein) and bile extract were all of porcine origin and obtained from Sigma-Aldrich (MO, USA). Amano lipase A (from fungus *Aspergillus niger*, 132,000 U/g) was provided by Amano Enzyme Inc. (IL, USA).

### Formation of nanoporous starch aerogel (NSA)

The NSA samples were produced from wheat starch according to the method of Ubeyitogullari and Ciftci using the optimized NSA formation conditions^29^. Briefly, wheat starch solution (10%, w/w) was gelatinized in a closed high-pressure reactor (4520 Bench Top Reactor, Parr Instrument Company, IL, USA) at 120 °C and 600 rpm for 20 min to obtain a hydrogel which was subsequently retrograded at 4 °C for 48 h. Then, the hydrogels were converted to alcogels with a five-step solvent exchange step by soaking the hydrogels in 30, 50, 70, and 100% (v/v) ethanol for 1 h, and in 100% ethanol for 24 h. Then the alcogels were converted to NSAs by removing the ethanol from the alcogels using SC-CO_2_ drying at 40 °C and 10 MPa for 4 h at a CO_2_ flow rate of 0.5 L/min (measured at ambient conditions) which were selected based on our previous study where the drying temperature, pressure and flow rate of CO_2_ were optimized for the highest surface area^29^. SC-CO_2_ drying of the alcogels was carried out in a custom-made laboratory scale SC-CO_2_ drying system which employed double head high pressure syringe pump (Model 260D, Teledyne Isco Inc., NE, USA) for pressurization. Details and operation of the SC-CO_2_ drying system were given somewhere else^28^.

### Simultaneous formation of low-crystallinity curcumin nanoparticles impregnated in the NSA

Formation of low-crystallinity curcumin nanoparticles impregnated into NSA was based on a modified NSA formation method described above. Excess amount of curcumin (1 g) was mixed with ethanol (40 mL) at room temperature (21 °C) and the undissolved curcumin was removed by filtration through a 0.45 µm pore-size filter. Then, the saturated curcumin solution was used in the last step of the solvent exchange described in the above section instead of 100% ethanol to obtain an alcogel called CUR-alcogel. Afterwards, CUR-alcogels were dried with SC-CO_2_ using the same system and conditions used in the formation of NSA to obtain curcumin impregnated NSA (CUR-NSA). In addition to ethanolic curcumin solution prepared at room temperature (21 °C), CUR-NSAs were prepared from ethanolic curcumin solution prepared at 60 °C using the same procedure. CUR impregnated NSAs were called CUR-NSA-60 °C or CUR-NSA-RT depending on the impregnation temperature of 60 °C or room temperature (RT), respectively. CUR-NSAs were stored in the freezer at −18 °C until analyzed.

### Morphology

The morphology of the NSA and the CUR-NSA was analyzed by field emission scanning electron microscope (S4700 FE-SEM, Hitachi, Tokyo, Japan) under low vacuum mode at 5 kV and 15 mA. The specimens were prepared by cutting thin cross-sections from the surface and the center of the NSA monoliths. The specimens were then sputter-coated with a chromium layer under vacuum (Desk V HP TSC, Denton Vacuum LLC, NJ, USA) prior to analysis.

The morphology of the curcumin in the bioaccessible fraction after simulated digestion (described in the simulated digestion section) was analyzed by transmission electron microscopy (H-7500 TEM, Hitachi, Tokyo, Japan) at an accelerating voltage of 80 kV. One drop of bioaccessible fraction was placed on 230 mm copper grids and air dried. Then, the samples were negatively stained with 1% phosphotungstic acid. After drying at room temperature (21 °C) for 8 h, the samples were examined by TEM. Furthermore, particle size of the curcumin particles in the NSA and in the bioaccessible fraction after simulated digestion was measured from the SEM and TEM images by examining the size of the randomly selected 50 particles using ImageJ v. 1.50i software, respectively.

### Crystallinity

Crystallinity of the CUR-NSA-RT, physical mixture of crude CUR with empty NSA (14.4 mg crude CUR/g empty NSA), CUR-NSA-60 °C, physical mixture of crude CUR with empty NSA (224.2 mg crude CUR/g empty NSA), empty NSA and crude CUR was studied with x-ray diffraction (XRD) analysis using a PANalytical Empyrean Diffractometer (Empyrean, PANalytical B.V., Almelo, Netherlands) equipped with a PIXcel^3D^ detector. The instrument was operated with 1D detection at 45 kV and 40 mA. The powdered samples were scanned from 2° to 40° (2*θ*) with a sampling interval of 0.05°.

### Fourier-transform infrared spectroscopy

The chemical interaction between the curcumin and the NSA was studied by Attenuated Total Reflectance Fourier-Transform Infrared Spectrometer (ATR-FTIR) (Nicolet 380, Thermo Scientific, MA, USA). FTIR spectroscopy was performed between 4000 and 400 cm^−1^ at spectral resolution of 4 cm^−1^ with 128 scans.

### Determination of the curcumin impregnation capacity

Curcumin was extracted from CUR-NSA (0.1 g) by acetonitrile at room temperature (21 °C) with occasional vortexing. Then, NSA was separated from the mixture by 0.45 µm pore-size filter. Finally, curcumin concentration in the filtrate was determined using HPLC as described in the curcumin analysis section. The impregnation capacity was reported as mg CUR/g NSA.

### Curcumin analysis

Curcumin was quantified by an HPLC (Agilent 1100 Series, Agilent Technologies, Germany) equipped with a variable wavelength detector (VWD) according to the method of Lungare *et al*.^45^ Briefly, an aliquot (20 µL) was injected onto a reversed phase Gemini C18 110 A column (150 × 4.6 mm, 5 µm; Phenomenex, CA, USA) that was maintained at 30 °C. The mobile phase consisted of acetonitrile and 5% acetic acid at a ratio of 45:55 (v/v) at a flow rate of 0.8 mL/min. The elution was monitored at 420 nm. Curcumin was quantified using an external calibration curve that was prepared using curcumin solutions at varying concentrations (0.1–50 µg/mL in acetonitrile).

### Simulated digestion

A simulated gastrointestinal digestion method was adopted from Minekus *et al*. to determine *in vitro* bioaccessibility of the curcumin^46^. Simulated salivary fluid (SSF), simulated gastric fluid (SGF) and simulated intestinal fluid (SIF) were prepared using a method reported previously^46^. Curcumin particles impregnated in the NSA and the physical mixture of crude curcumin with empty NSA (14.4 mg crude CUR/g empty NSA) was used as a control. All digestion experiments were carried out in triplicate.

#### Oral phase

First, the curcumin impregnated NSA sample (0.25 g) and SSF electrolyte stock solution (3.5 mL) were added into a flask. Then, α-amylase solution (0.5 mL, 750 U/mL) was included. Subsequently, 0.3 M CaCl_2_ (25 µL) and deionized water (0.975 mL) were added. Finally, pH of the mixture was adjusted to pH 7.0 and the mixture was agitated in a shaking water bath for 30 sec at 37 °C and 150 rpm^46,47^.

#### Gastric phase

After the oral digestion, oral bolus (5 mL) was mixed with SGF electrolyte stock solution (3.25 mL, pH 3.0) and the pH was adjusted to 3.0 using 1 M HCl (75 µL). Afterwards, porcine pepsin solution (0.5 mL, 40 000 U/mL) and fungal lipase (0.25 mL, 1000 U/mL) were added. There is no commercial gastric lipase and therefore fungal lipase was included as an analogue to human gastric lipase^48^. Then, 0.3 M CaCl_2_ (2.5 µL) and deionized water (0.923 mL) were included. The final mixture was incubated at 37 °C and 100 rpm for 2 h.

#### Intestinal phase

After the gastric digestion, SIF electrolyte stock solution (6.125 mL, pH 7.0) was mixed with the gastric chyme (10 mL). Then, pancreatin solution (1.25 mL) was prepared in SIF electrolyte stock solution according to α-amylase activity and added into the mixture. Moreover, extra porcine pancreatic lipase was added to achieve a lipase activity of 2000 U/mL. Then, fresh bile solution (0.625 mL, 320 mM, prepared in SIF), 0.3 M CaCl_2_ (20 µL) and deionized water (1.95 mL) were added to the mixture and the pH was adjusted to pH 7.0 using 1 M HCl (30 µL). Lastly, the final mixture was incubated at 37 °C and 100 rpm for 2 h.

#### Bioaccessible fraction

The bioaccessible fraction after digestion was separated using the method of Alemany *et al*.^49^ Immediately after simulated digestion experiments, the flasks were placed into an ice bath to stop digestion. Then, bioaccessible fraction of the digested samples was attained by centrifugation at 4 °C at 4000 rpm for 90 min (Allegra X-15R, Beckman Coulter, CA, USA). The bioaccessibility (%) of curcumin was calculated as follows:

1 $$Bioaccessibility\,( \% )=\frac{Curcumin\,in\,the\,bioaccessible\,fraction}{Total\,curcumin\,included}\times 100$$

The concentration of curcumin in the bioaccessible fraction was determined using the HPLC method described above. The samples were filtered through 0.45 µm pore-size filter prior to analysis.

### Statistical analysis

Statistical analysis of the obtained data was performed using Minitab® 16.1.1 software (Minitab Inc., State College, PA, USA). Tukey’s multiple comparison test was applied and the differences among treatments were considered to be statistically significant when p < 0.05.
